# Epidemiology of Pediatric Acute Respiratory Distress Syndrome in Singapore: Risk Factors and Predictive Respiratory Indices for Mortality

**DOI:** 10.3389/fped.2014.00078

**Published:** 2014-07-25

**Authors:** Judith Ju-Ming Wong, Tsee Foong Loh, Daniela Testoni, Joo Guan Yeo, Yee Hui Mok, Jan Hau Lee

**Affiliations:** ^1^Department of Pediatric Medicine, KK Women’s and Children’s Hospital, Singapore, Singapore; ^2^Children’s Intensive Care Unit, Department of Pediatric Subspecialties, KK Women’s and Children’s Hospital, Singapore, Singapore; ^3^Duke-NUS Graduate School of Medicine, Singapore, Singapore; ^4^Division of Neonatal Medicine, Escola Paulista de Medicina, Universidade Federal de São Paulo, São Paulo, Brazil

**Keywords:** acute respiratory distress syndrome, acute lung injury, critical care, children, pediatric intensive care unit, epidemiology, pulse oximetry, Asia

## Abstract

**Aim:** Acute respiratory distress syndrome (ARDS) represents the most severe form of acute lung injury. The aim of our study is to describe the epidemiology of pediatric ARDS in Singapore and compare the outcomes of ARDS using the following respiratory indices: P_a_O_2_/F_i_O_2_ ratio (P/F ratio), S_p_O_2_/F_i_O_2_ ratio (S/F ratio), oxygenation index (OI), and oxygen saturation index (OSI).

**Methods:** We examined medical records of patients admitted to the Children’s Intensive Care Unit in KK Women’s and Children’s Hospital from 2009 to 2012. Those who fulfilled criteria for the American-European Consensus Conference definition for ARDS were identified. Demographic, clinical, and radiographic information were extracted and analyzed.

**Results:** We identified 70 patients with ARDS. Median age (interquartile range) was 6.2 (1.4, 10.4) years. The most common risk factor was pneumonia [50 (71%)]. Overall mortality was 44 (63%) patients. Thirty-two (56%) patients had an underlying chronic comorbidity; 18 (46%) were hematology–oncology conditions. Fifty-six (80%) patients had multiorgan dysfunction. Adjunct therapies used in our patients included inhaled nitric oxide [5 (7%)], prone position [22 (31%)], steroids [26 (37%)], and neuromuscular blockade [26 (37%)]. A high OI and low PF ratio after 24 h of diagnosis of ARDS were associated with mortality. From day 3 onward, all four respiratory indices appropriately differentiated survivors from non-survivors. Severity based on the S/F ratio and OSI demonstrated association with decreased ventilator free days and ICU free days.

**Conclusion:** Risk factors for mortality included having an underlying comorbidity, multiorgan dysfunction, a low PF ratio, and high OI at 24 h of ARDS. Abnormal S_p_O_2_-based measurements were reliable markers of poor outcomes in pediatric ARDS.

## Introduction

Acute respiratory distress syndrome (ARDS) represents the most severe form of acute lung injury (ALI) and is characterized by alveolar leukocyte infiltration and protein-rich pulmonary edema (1). Compared with those without lung injury, children with ALI/ARDS have significant increased morbidity and mortality in the pediatric intensive care unit (PICU) (1, 2).

Compared to adults, the incidence of ARDS is lower in children (1–4% of all PICU admissions) (3). Mortality from pediatric ARDS varies widely between 20 and 62% (1, 2, 4–8). In contrast to data from adults where mortality from ARDS has declined from approximately 64–70% in the 1980s to 29–42% in the twenty-first century, similar trends have not been demonstrated in the pediatric literature (9–13). The epidemiology of pediatric ARDS in Singapore has not been previously described.

The most well accepted definition for pediatric ARDS is extrapolated from the adult definition based on the American-European Consensus Conference (AECC), which utilizes the arterial partial pressure of oxygen (P_a_O_2_) to fraction of inspired oxygen (F_i_O_2_) (P/F) ratio (14, 15). Another P_a_O_2_-based index, oxygenation index (OI), has the inherent advantage over P/F ratio through the incorporation of the mean airway pressure (MAP). A higher OI in itself has been shown to independently predict mortality (1, 16–19). Due to challenges in placing arterial catheters to periodically measure the P_a_O_2_ in children, investigators have utilized and validated respiratory indices using non-invasive oxygen saturation (S_p_O_2_)-based measurements (14, 20).

The primary aim of this study is to describe the epidemiology of pediatric ALI/ARDS in Singapore. The optimal variable to quantify oxygenation impairment for risk stratification remains uncertain (20). Therefore, our study’s secondary aim is to compare the outcomes of ALI/ARDS using both P_a_O_2_-based indices (P/F ratio and OI) and S_p_O_2_-based indices [S_p_O_2_/F_i_O_2_ ratio (S/F ratio) and oxygen saturation index (OSI)].

## Materials and Methods

### Patients

We collected data on all patients admitted to our Children’s Intensive Care Unit (CICU) over a 4-year period (January 1, 2009 to December 31, 2012). Our hospital is one of two tertiary pediatric centers in Singapore that receives national and regional pediatric referrals. Our CICU is a 16-bedded multidisciplinary facility that admits medical, surgical, and cardio-thoracic patients. This study has been approved by our hospital’s institutional review board and a waiver of consent was granted.

In our institution, all hospitalized patients are coded using either the International Classification of Diseases [ICD9CM or ICD10AM (from 2012 onward)] or the SCT (SNOMED CLINICAL TERMINOLOGY) code. We generated a patient list with ALI/ARDS using these codes. We then examined patients’ charts to confirm that these patients fulfilled criteria for the AECC’s definition for ALI/ARDS. In addition, an independent search of the CICU’s admission log was also performed, identifying all patients that fulfilled the AECC definition of ALI/ARDS, regardless whether they had been coded as such, to ensure a complete pick up rate.

We included all patients aged 1 day to 16 years of age that had either a primary or secondary diagnosis of ALI/ARDS in their discharge or death summaries. Premature neonates (gestational age <35 weeks), inborn neonates, and patients being cared for in the neonatal intensive care unit (NICU) were excluded.

### Data collection

Demographic, clinical, and radiographic data were extracted through chart review using a standardized data collection form. Severity of illness scores including the PRISM 2 and PELOD score were taken on PICU admission. We recorded arterial blood gas measurements and ventilator modes on day of diagnosis, 24 h, day 3, and day 7 of ALI/ARDS. We also collected data on adjunct therapies used for ARDS including inhaled nitric oxide, prone position, steroids and neuromuscular blockade, as well as, the use of other support modalities [e.g., inotropes, continuous renal replacement therapy, and extracorporeal membrane oxygenation (ECMO)]. Positive blood cultures were also recorded.

### Definitions

We defined mortality as all deaths occurring during the CICU stay. To account for death as a competing outcome, we summarized the requirement for mechanical ventilation as being free and alive from mechanical ventilation with a maximum of 28 days from the diagnosis of ALI/ARDS [28-ventilator free days (VFD)]. Similarly, in addition to length of CICU stay, we also calculated CICU-free days with a maximum of 28 days [28-ICU free days (IFD)]. Steroid exposure was defined as any use of systemic corticosteroids. We assessed for organ dysfunction on day 7 of ARDS or death, whichever was earlier. We utilized the International Pediatric Sepsis Consensus Conference 2005 criteria for organ dysfunction (21). We defined multiorgan dysfunction when two or more extra-pulmonary organ systems were involved. We considered any pneumothorax, pneumomediastinum, or pneumopericardium as respiratory complications from mechanical ventilation. Cut offs for ALI and ARDS for the four respiratory indices are summarized in Table 1.

**Table 1:** **Respiratory indices used to define ALI/ARDS**.

Index	ALI criteria	ARDS criteria	
P/F ratio	200–300 (mild)^a^	<200 (moderate)^a^	<100 (severe)^a^
S/F ratio	212–253	<212
OI^b^	5.3–8.1	>8.1
OSI^c^	6.5–7.8	>7.8

*ALI – acute lung injury*.

*ARDS – acute respiratory distress syndrome*.

*P/F ratio – partial pressure of arterial oxygen (P_a_O_2_): fraction of inspired oxygen ratio (F_i_O_2_)*.

*S/F ratio – oxygen saturation (S_p_O_2_): fraction of inspired oxygen ratio*.

*^a^ Berlin definition sub-classifies ARDS to mild, moderate, and severe based on the P/F ratio*.

*^b^ OI – oxygenation index = mean airway pressure × F_i_O_2_/P_a_O_2_*.

*^c^ OSI – oxygen saturation index = mean airway pressure × F_i_O_2_/S_p_O_2_*.

### Statistical analysis

Categorical data were presented as counts and percentages and continuous data were presented as medians and interquartile ranges (IQR). Differences between categorical variables were analyzed using the chi-square test or Fisher’s exact test, whichever was appropriate. Differences between continuous variables were analyzed using the Wilcoxon’s signed rank test. Statistical analysis was performed using Stata 13.1 (College Station, TX, USA). All statistical tests were two-tailed and the significance level was taken as *p* < 0.05. Risk factors for mortality were corrected for PRISM 2 scores using bivariate analysis. We did not perform the bivariate analysis for P/F ratio and OI because the components of these indices are also part of the PRISM 2 score.

## Results

We had a total 4079 CICU admissions between years 2009 and 2012. Of these, 70 patients (17.2 per 1000 CICU admissions or 1.7%) fulfilled the AECC’s criteria for ALI/ARDS (Table 2). The median age of our cohort was 6.0 (1.4, 10.4) years. The overall median of PRISM 2 and PELOD score was 12 (7, 17) and 12 (1, 22), respectively. The common risk factors included pneumonia, which was present in 50 (71%) of patients and sepsis, which was present in 38 (54%) of patients. Overall, at diagnosis of ALI/ARDS, our cohort had a median P/F ratio of 112.6 (71.6, 147.8), S/F ratio of 130.5 (100.0, 163.3), OI of 17.3 (11.3, 33.3), and OSI of 21.1 (12.9, 29.0).

**Table 2:** **Characteristics of pediatric patients with ALI/ARDS**.

Characteristic	Survivors (*n* = 26)	Non-survivors (*n* = 44)	*P*-value
Age (years)	4.4 (1.0, 8.8)	7.7 (1.4, 11.1)	0.331
Age category
<2 years	7 (35%)	13 (65%)	0.670
2–12 years	15 (44%)	21 (56%)	
>12 years	4 (29%)	10 (71%)	
Weight (kg)	15.8 (10.0, 25.0)	18.0 (8.7, 35.5)	0.589
PRISM 2 score	8.5 (4.5, 14.8)	12.5 (7, 20.0)	0.121
PELOD score	11.5 (1, 12.0)	12 (1, 24.8)	0.156
Multiorgan dysfunction^a^	16 (62%)	40 (91%)	0.008*
Presence of comorbidities	8 (31%)	31 (70%)	0.001*
Neuromuscular	1	6	
Cardiovascular	0	4	
Respiratory	1	6	
Renal	0	0	
Gastrointestinal	0	2	
Hematology–oncology	2	16	
Metabolic	1	0	
Genetic/congenital	3	2	

*Values are in median (interquartile range) and counts (percentages)*.

*PRISM 2 – Pediatric risk of mortality*.

*PELOD – Pediatric logistic organ dysfunction*.

^a^ Defined as two or more extra-pulmonary organ dysfunction

*Statistically significant (*p* < 0.05).

The overall median length of ICU and hospital stay was 10 (4, 19) and 19 (7, 57) days, respectively. The median IFD was 0 (0, 10) days. Forty-four (63%) patients in our cohort died and 4 (5.7%) required ECMO support. The overall median duration of ventilation was 10 (4, 21) days and overall median VFD was 0 (0, 13) days. Thirty-nine (56%) patients had a chronic comorbidity and 56 (80%) had multiorgan dysfunction. Presence of comorbidities and multiorgan dysfunction were associated with mortality even after adjusting for severity of illness (Table 2).

**Figure 1:**
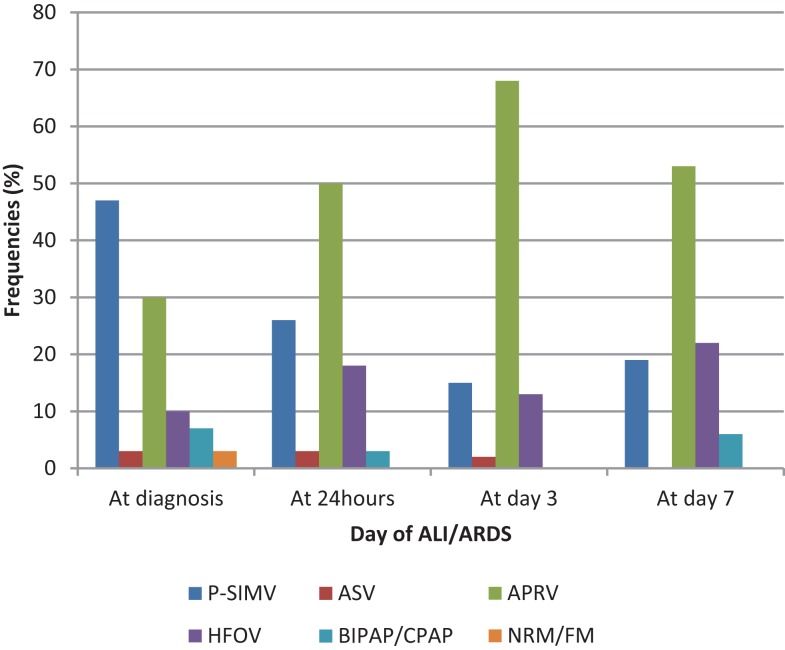
**Relative frequencies of modes of ventilation during the first week of ALI/ARDS are shown**. P-SIMV: pressure-synchronized intermittent mandatory ventilation. ASV: adaptive support ventilation, APRV: airway pressure release ventilation. HFOV: high-frequency oscillatory ventilation. BIPAP: bi-level positive airway pressure. CPAP: continuous positive airway pressure. NRM: non-rebreather mask oxygen. FM: face mask oxygen.

A total of 7 (10%) patients did not require invasive mechanical ventilation at the onset of ALI/ARDS. Among those that required invasive mechanical ventilation, the most common mode of ventilation at diagnosis was pressure-synchronized intermittent mandatory ventilation (P-SIMV) [32 (47%)] followed by airway pressure release ventilation (APRV) [21 (30%)]. However, the mode of ventilation changed over the course of illness with increasing usage of APRV and high-frequency oscillatory ventilator (HFOV) after 24 h of diagnosis of ALI/ARDS (Figure 1).

Adjunct therapies that were used included inhaled nitric oxide, prone position, steroids, and neuromuscular blockade (Table 3). In patients who required the use of neuromuscular blockade, the median duration of therapy was 4.5 (3.0, 9.0) days. Steroid exposure occurred in 26 (37%) patients for various indications including refractory shock (*n* = 7), respiratory (e.g., pneumonitis, chronic lung disease, and fibrotic stage ARDS) (*n* = 12), and other (e.g., pre-medications and drug reactions) (*n* = 7).

**Table 3:** **Use of adjunct therapies for ARDS and other supportive therapies**.

Adjunct therapies/supportive therapies	Survivors (*n* = 26)	Non-survivors (*n* = 44)	*P*-value
iNO	2	3	0.619
Prone	10	12	0.330
Steroid exposure	5	21	0.017*
Neuromuscular blockade	10	16	0.861
Inotropes	19	38	0.167
CRRT	3	7	0.449
ECMO	3	1	0.141

*ARDS – acute respiratory distress syndrome*.

*iNO – inhaled nitric oxide*.

*CRRT – continuous renal replacement therapy*.

*ECMO – extracorporeal membrane oxygenation*.

*Statistically significant (*p* < 0.05).

Sixteen (23%) patients had positive blood cultures and the commonest organism was *streptococcus* sp. (*n* = 4) followed by *staphylococcus* sp. (*n* = 3) and *pseudomonas aeruginosa* (*n* = 3). A total of 15 (21%) patients had complications of air leak including pneumothorax, pneumomediastinum, and pneumopericardium.

There was no significant difference between all the respiratory indices at diagnosis of ALI/ARDS among survivors and non-survivors. However, both P_a_O_2_-based indices (high OI and low P/F ratio) after 24 h of diagnosis of ARDS onward were associated with mortality but not S_p_O_2_-based indices. From day 3 onward, all four indices were able to differentiate patients with increased mortality (Table 4).

**Table 4:** **Respiratory indices at the onset, after 24 h, on day 3, and on day 7 of diagnosis of ALI/ARDS**.

Respiratory indices	Survivors (*n* = 26)	Non-survivors (*n* = 44)	*P*-value
**AT DIAGNOSIS**			
P/F ratio	123 (92,148)	107 (65,154)	0.258
S/F ratio	138 (116,180)	121 (92,162)	0.175
OI	16 (14,27)	19 (11,34)	0.375
OSI	19 (13,30)	22 (13,28)	0.764
**AT 24 h**			
P/F ratio	160 (124,255)	103 (63,188)	0.011*
S/F ratio	192 (140,222)	137 (99,218)	0.071
OI	12 (7,23)	23 (10,41)	0.038*
OSI	12 (8,18)	18 (9,29)	0.145
**AT DAY 3**			
P/F ratio	180 (123,276)	106 (60,212)	0.006*
S/F ratio	221 (170,303)	149 (91,243)	0.006*
OI	12 (6,17)	25 (7,53)	0.022*
OSI	10 (7,13)	18 (8,33)	0.010*
**AT DAY 7**			
P/F ratio	219 (155,293)	124 (66,226)	0.010*
S/F ratio	277 (175,330)	146 (102,222)	0.002*
OI	8 (6,13)	22 (8,49)	0.016*
OSI	7 (6,12)	20 (9,30)	0.013*

*Values are in median (interquartile range)*.

*ALI – acute lung injury*.

*ARDS – acute respiratory distress syndrome*.

*P/F ratio – partial pressure of arterial oxygen: fraction of inspired oxygen ratio*.

*S/F ratio – oxygen saturation: fraction of inspired oxygen ratio*.

*OI – oxygenation index*.

*OSI – oxygenation saturation index*.

*Statistically significant (*p* < 0.05).

S_p_O_2_-based indices were associated with VFD and IFD, a low S/F ratio at diagnosis was associated with decreased VFD and IFD, and a high OSI at 24 h of ARDS was associated with decreased IFD. There was no association between P_a_O_2_-based indices (P/F ratio and OI) with VFD and IFD.

## Discussion

Our study demonstrated that ALI/ARDS occurred in approximately 2% of all CICU admissions. Overall mortality remained very high. This study highlighted the following: (1) there was a trend toward the use of alternative modes of mechanical ventilation (e.g., APRV, HFOV); (2) a lower P/F ratio and higher OI after 24 h of diagnosis of ARDS were associated with increased mortality; and (3) SpO_2_-based measurements were as reliable as P_a_O_2_-based indices to quantify oxygenation failure (especially after 3 days of ARDS onset).

There are several similarities between our study’s findings and those reported previously. Our incidence of ALI/ARDS of 1.7% of all CICU admissions was consistent with previous reports (0.6–7%) (1, 2, 4–6). The causes and risk factors of ARDS in our population were also similar to other reports and predominantly involved pneumonia and sepsis (1, 22). The need for ECMO support in our cohort was 5.7% (*n* = 4), which was similar to other centers in Australia/New Zealand (3.4%) (1). However, some centers in France reported the use of ECMO to be as high as 22% (2).

The CICU mortality rate from ALI/ARDS in our study (63%) was higher compared with other pediatric ARDS reports (20–35%) (1, 5, 6, 8, 22). However, there are scattered reports of higher mortality numbers ranging from 60 to 63% (2, 4, 23). The high mortality rates in our cohort can be attributed to the fact that a large majority (91%) of our patients satisfied the ARDS criteria (P/F ratio <200) rather than ALI (P/F ratio 200–300) at diagnosis. In addition, more than half of our cohort also had an underlying chronic comorbidity (56%). Among those with comorbidities, hematology–oncology diagnoses were the most common (46%). Pre-existing chronic organ dysfunction and immunosuppression have been associated with increased mortality in pediatric ALI/ARDS (1, 2, 23). Excluding hematology–oncology patients, mortality rate in our cohort was 53.8%. Moreover, 80% of our cohort had multiorgan dysfunction.

The most common initial mode of ventilation was pressure controlled ventilation. This reflects the mode with which most pediatric intensivists are traditionally comfortable with (22, 24). This utility of this mode, however, steadily declined as the disease progressed and other modes of ventilation were adopted. HFOV has been used to improve oxygenation in pediatric patients with ARDS and severe hypoxemia refractory to conventional ventilatory support (25–27). The evidence for APRV specifically in improving oxygenation in pediatric ARDS is growing; however, the only ventilation strategy that has been conclusively associated with improved outcomes is the use of low tidal volumes (23, 28–30). Till date, there are no randomized controlled trials comparing APRV to other modes of ventilation.

The P/F ratio features prominently in multiple definitions for ARDS including the AECC definition, the Berlin definition, the Murray lung injury score (or Pediatric lung injury score), and the Delphi consensus panel definition (31–34). This index requires arterial blood gas sampling, which is difficult to obtain in some children, thus rendering the P/F ratio problematic for early diagnosis and monitoring. Indeed, a small number of the patients in our cohort had missing data due to failure in obtaining arterial samples [10/280 (3%) of blood gas samples were capillary samples and were excluded from the analysis]. The use of the S/F ratio in ARDS diagnosis was previously described in two pediatric trials (14, 20). The oxyhemoglobin dissociation curve is nearly linear when S_p_O_2_ is between 80 and 97%. Subsequently, it levels off such that a large increment in P_a_O_2_ produces only a tiny increment in S_p_O_2_. Therefore, it may be possible to characterize pediatric ALI/ARDS accurately by substituting S_p_O_2_ for P_a_O_2_ at levels ≤97% (35). The cutoffs for S/F ratio and OSI for diagnosis of ALI/ARDS are summarized in Table 1. From our data, an S/F ratio and OSI within the ARDS category was significantly associated with decreased VFD and IFD. Our data add evidence that S_p_O_2_-based measurements were reliable in differentiating patients with poorer outcome. The value of having these non-invasive tools to screen for ARDS is to enable early recognition in clinical practice, early institution of proven therapies, allow prediction of outcomes, and lastly facilitate clinical research or epidemiologic studies (20, 36).

The OI has an inherent advantage over the P/F ratio used in that it incorporates MAP. A higher OI in itself has been shown to independently predict mortality (1, 16–19). This was also demonstrated in our study (Table 4). Other centers have used a low OI as criteria for extubation and a high OI for use of HFOV and ECMO (37, 38). In agreement with previous studies, we found that the impact of the severity of ARDS on outcome as represented by the OI was found to be less predictable within the first 24 h after diagnosis but was consistent thereafter (16). A P/F ratio within the ARDS range was also associated with mortality after 24 h of diagnosis. It is very likely that in the early phase of ARDS, therapeutic interventions, such as recruitment maneuvers and the use of optimal positive end expiratory pressure to restore functional residual capacity, as well as response to specific treatment will cause significant improvement in OI and P/F ratio in a subset of children with ARDS.

The strength of this study is that all CICU admissions were individually screened according to the AECC criteria for ARDS to ensure a complete pick up rate. This is also the first study to describe pediatric ARDS in Singapore, which adds to the limited known epidemiology of ARDS within Southeast Asia (4, 39). There are several limitations to this study. While all attending physicians in our CICU targeted 6 ml/kg of tidal volume and limiting peak inspiratory pressures to below 30 cm H_2_O on conventional mechanical ventilation, there was no strict protocol in our unit for switching between conventional and alternative modes of ventilation; this was left to the discretion of the attending physicians on duty. We found a statistical association between steroid exposure and increased mortality in pediatric ARDS (Table 3). However, the indications and dosages for steroid use were not standardized in our cohort of patients. Moreover, the retrospective nature of this study precludes us from making any cause-and-effect association.

## Conclusion

This first description of pediatric ARDS in Singapore demonstrated a high overall mortality. Risk factors for mortality include presence of comorbidities, multiorgan dysfunction, low PF ratio, and high OI at 24 h of ARDS. Abnormal S_p_O_2_-based measurements were reliable alternative markers of poor outcomes in pediatric ARDS.

## Author Contributions

Judith Ju-Ming Wong, Tsee Foong Loh, Jan Hau Lee: conception and design of study. Judith Ju-Ming Wong: acquisition of data. Judith Ju-Ming Wong, Tsee Foong Loh, Daniela Testoni, Yee Hui Mok, Joo Guan Yeo, Jan Hau Lee: analysis and interpretation of data. Judith Ju-Ming Wong, Tsee Foong Loh, Daniela Testoni, Yee Hui Mok, Joo Guan Yeo, Jan Hau Lee: drafting and critically revising manuscript.

## Conflict of Interest Statement

The authors declare that the research was conducted in the absence of any commercial or financial relationships that could be construed as a potential conflict of interest.
